# Comparative studies on speciation: 30 years since Coyne and Orr

**DOI:** 10.1111/evo.14181

**Published:** 2021-02-18

**Authors:** Daniel R. Matute, Brandon S. Cooper

**Affiliations:** ^1^ Biology Department University of North Carolina Chapel Hill North Carolina 27510; ^2^ Division of Biological Sciences University of Montana Missoula Montana 59812

**Keywords:** Speciation, Haldane's rule, Postyzygotic isolation

## Abstract

Understanding the processes of population divergence and speciation remains a core question in evolutionary biology. For nearly a hundred years evolutionary geneticists have characterized reproductive isolation (RI) mechanisms and specific barriers to gene flow required for species formation. The seminal work of Coyne and Orr provided the first comprehensive comparative analysis of speciation. By combining phylogenetic hypotheses and species range data with estimates of genetic divergence and multiple mechanisms of RI across *Drosophila*, Coyne and Orr's influential meta‐analyses answered fundamental questions and motivated new analyses that continue to push the field forward today. Now 30 years later, we revisit the five questions addressed by Coyne and Orr, identifying results that remain well supported and others that seem less robust with new data. We then consider the future of speciation research, with emphasis on areas where novel methods and data motivate potential progress. While the literature remains biased towards *Drosophila* and other model systems, we are enthusiastic about the future of the field.

Speciation is the process in which populations diverge into groups that cease to exchange alleles. A crucial aspect of speciation is the development of reproductive isolating barriers that reduce gene flow between incipient species, although reproductive isolation (RI) is not essential for divergence (Turelli et al. 2001; Nosil 2008). The connection between speciation and the evolution of RI was championed by both Dobzhansky (Dobzhansky 1937) and Mayr (1942), but was earlier developed by Poulton (1904) and Wallace (1865; both reviewed in Mallet 2004). Evolutionary geneticists have characterized reproductive isolating barriers for over 100 years (Dobzhansky 1937; Mayr 1963; Coyne and Orr 2004; Sobel et al. 2010; Nosil 2012), focusing on when barriers occur in the reproductive cycle. Prezygotic barriers occur before a zygote is formed and include ecological barriers (e.g., habitat differences, Sobel et al. 2010), behavioral barriers (e.g., signaling differences, Wilkins et al. 2013; Schaefer and Ruxton 2015), mechanical barriers (e.g., genitalia differences, Grant 1994; Sota and Tanabe 2010), and gametic incompatibilities (Howard 1999). Barriers that occur after fertilization but before a zygote is formed are called postmating‐prezygotic (PMPZ; Howard 1999; Coyne and Orr 2004). Postzygotic barriers occur after a hybrid zygote is formed and include phenotypes as extreme as hybrid sterility and inviability (Orr and Presgraves 2000; Orr 2005; Orr et al. 2007), but also more nuanced traits, such as hybrid behavioral defects (Turissini et al. 2017; McQuillan et al. 2018) or delays in development (Burton 1990; Matute and Coyne 2010). Understanding the evolution of RI is crucial to explain how variation within populations is converted to variation between populations to generate species.

Meta‐analyses have been a productive avenue to study the speciation process. Early studies evaluated the extent of developmentally‐based (intrinsic) postzygotic isolation in groups with different levels of differentiation (Zouros 1973; Ayala et al. 1974; Wilson et al. 1974; Prager and Wilson 1975). While Zouros (1973) found no correlation between genetic distance and the degree of postzygotic isolation, Ayala found that higher taxonomic units showed more isolation than lower units. But it was the seminal comparative analysis of Coyne and Orr (1989) (hereafter, “C&O”) that combined phylogenetic hypotheses with range data, divergence estimates, and empirical estimates of premating and intrinsic postzygotic isolation to rigorously test some of the most important questions in speciation research for the first time. By using published data from 119 *Drosophila* interspecific hybridizations and their genetic distances, C&O (1989) was the first study to incorporate measurements of hybrid laboratory fitness with molecular divergence.

The importance of C&O (1989) on the field of speciation research is hard to overstate. C&O (1989) gave rise to a cottage industry of similar meta‐analyses, each focused on particular aspects of the speciation process. Researchers have and continue to re‐analyze the data that C&O (1989) assembled to ask specific questions about speciation. These include analysis of how and when Haldane's rule (i.e., the absence, rarity, or sterility of the heterogametic sex in interspecific crosses) appears (Turelli and Begun 1997), correlations between pre‐ and postmating isolation with allozyme and silent DNA divergence (Fitzpatrick 2002), and the influence of ecological divergence on prezygotic and intrinsic postzygotic RI evolution (Funk et al. 2006). These data have also been used to test for reinforcing natural selection on prezygotic isolation in areas of sympatry as a response to maladaptive hybridization (Dobzhansky 1937; Dobzhansky 1940; Blair 1955). Analyses of reinforcement have focused on the role of range overlap (Nosil 2013) and “concordant isolation asymmetries” (Yukilevich 2012), in addition to the relative roles of intrinsic postzygotic isolation, ecological differentiation, and *X* chromosome size in speciation (Turelli et al. 2014). C&O themselves revisited these data, adding an additional 52 interspecific *Drosophila* hybridizations to their original dataset (Coyne and Orr 1997). Now 30 years later, we revisit the impact of C&O (1989) on the field.

## The Five Original Questions

C&O (1989) addressed five core questions about the speciation process in *Drosophila*: (i) how rapidly does RI evolve, (ii) do prezygotic and postzygotic isolation evolve at the same rate, *iii*) do hybrid sterility and inviability evolve at the same rate, *iv*) how does postzygotic isolation increase with time, and *v*) is prezygotic isolation enhanced by natural selection when populations become sympatric? In the last 30 years since the publication of C&O (1989), these five questions have become an integral part of the field of speciation biology. We revisit each question, describing the progress made and the importance of the results to the field. We also propose future directions, including limitations that are likely to limit progress in some areas. Even though C&O (1989)’s original piece was published just over 30 years ago, the approaches, the datasets, and the concepts they proposed still spark controversy and remain as relevant today as they were at the time of publication.

### HOW RAPIDLY DOES RI EVOLVE?

C&O stated that “the divergence time of taxa must obviously be correlated with the amount of reproductive isolation between them, because all species begin as populations that are not reproductively isolated”. In spite of some earlier precedents (Zouros 1973; Ayala et al. 1974), C&O (1989) was the first study to demonstrate this correlation using data on the magnitude of RI and pairwise genetic distance compiled by them and others (Bock 1984). Importantly, C&O (1989) incorporated phylogenetic corrections to the study of RI, which is crucial given that amounts of isolation among species pairs may not be evolutionarily independent due to their relatedness (Huey and Pianka 1981; Felsenstein 1985). C&O (1989) relied on published phylogenies (Throckmorton 1975, 1982; MacIntyre and Collier 1986) and a procedure inspired by Felsenstein (1985) to correct their data (reviewed in Huey et al. 2019), allowing only one comparison between the species on either side of a phylogenetic bifurcation (Fig. 3 in C&O). This phylogenetic correction ultimately reduced the data from 119 interspecific hybridizations to 42.

C&O (1989) found a monotonic increase in both prezygotic and postzygotic RI as divergence increases between species (Figs. 2 and 4 in C&O), a pattern that has been repeatedly tested and supported across divergent taxa (reviewed in Edmands 2002; Gourbière and Mallet 2010; Coughlan and Matute 2020). In addition to *Drosophila* (Coyne 1989; Coyne and Orr 1997; Turissini et al. 2018), the pattern of rapid behavioral evolution seems to apply to *Etheostoma* fish (Mendelson 2003) and *Desmognathus* salamanders (Tilley et al. 1990). Few examples have addressed the rate of evolution of the particular traits involved in prezygotic isolation. In Mormyd fish, differentiation in sexual cues (electric signals) increases with phylogenetic divergence and usually outpaces differentiation in ecologically important traits (Arnegard et al. 2010). Similarly, in Australian field cricket species (*Teleogryllus* spp.), male song becomes more differentiated as divergence accrues (Moran et al. 2020). In *Etheostoma* fish male conspecific preference appears at lower genetic distances than female conspecific preference (Mendelson et al. 2018). This pattern of increasing trait differentiation with genetic distance is not universal. The magnitude of interspecific differences in courtship song in *Drosophila* (Gleason and Ritchie 1998), and bird plumage coloration (Campagna et al. 2012; Moran et al. 2017) does not increase as divergence accrues. In cichlids, for example, assortative mating is more correlated with ecological niche and morphology than with genetic distance (Stelkens and Seehausen 2009). Since premating isolation is often the result of multimodal signaling (e.g., Ritchie and Gleason 1995; Nosil and Hohenlohe 2012), it is likely that no individual trait will show an increase over time, but the combination of traits might.

More support exists for increasing postzygotic RI with genetic distance, possibly because more taxa have been sampled. Multiple reviews have compiled cases of postzygotic isolation across divergence (Edmands 2002; Gourbière and Mallet 2010; Coughlan and Matute 2020), finding that a monotonic increase in the strength of hybrid inviability and sterility is common. However, there are also exceptions to the rule; for example, hybrid inviability in darters shows no systematic increase with genetic distance, caused in part by the occurrence of hybrid vigor at intermediate levels of divergence (Mendelson 2003). In stalk‐eyed flies hybrid male and female sterility increase with genetic distance but male hybrid inviability does not (Charistianson et al. 2005). Prezygotic isolation in orchids showed no increase over genetic distance for different reasons. While premating isolation in food‐deceptive orchids and PMPZ in food‐ and sexually‐deceptive orchids show variation but no correlation with divergence, sexually deceptive orchids display universally strong premating isolation since very early stages of divergence (Scopece et al. 2007; but see Sobel and Randle 2009 and Scopece et al. 2009). While increasing RI with genetic distance is not universal, the majority of cases suggest a positive correlation between the strength of RI and the genetic distance between species pairs.

While a primary focus since as early as the 19th century (Darwin 1859, Ch. 8 pp. 245–278), hybrid sterility and inviability are not the only forms of postzygotic RI. A broad range of traits can result in reduced hybrid fitness, including aberrant hybrid migratory behavior (Delmore and Irwin 2014), transgressive mating behavior (Gottsberger and Mayer 2007; Clark et al. 2010; Kost et al. 2016), decreased attractiveness (Naisbit et al. 2001; Lemmon and Lemmon 2010; Serrato‐Capuchina et al. 2020), and lower ability to locate a suitable substrate (Linn et al. 2004; Bendall et al. 2017; Turissini et al. 2017). Some evidence suggests that the likelihood that hybrids show transgressive phenotypic values in traits associated with species recognition increases as parental divergence between parentals increases (Stelkens et al. 2009). Phenotypic mismatch in hybrids may also generate postzygotic isolation (McBride and Singer 2010; Singer and McBride 2010; Arnegard et al. 2014; Cooper et al. 2018). For example, hybrids produced by *D. yakuba* and the forest species *D. teissieri* on the island of Bioko in West Africa prefer warm and dry habitats like *D. yakuba*, but they have low desiccation tolerance like *D. teissieri*, leaving them physiologically ill equipped to perform in their chosen habitat (Cooper et al. 2018). While these studies demonstrate that other postzygotic defects may exist in hybrids, the rates at which they evolve remain understudied.

Ecologically based postzygotic isolation and behavioral defects both increase with divergence, but these studies are substantially rarer than those involving hybrid inviability and hybrid sterility. Habitat isolation in either its premating (e.g., habitat divergence) or postzygotic (e.g., hybrid inviability; Funk et al. 2006) forms, ability to colonize hosts (Vienne et al. 2009), and subtle forms of postzygotic isolation (Turissini et al. 2017) also accrue with genetic distance. To date, most studied barriers to gene flow increase with divergence, albeit their rates of accumulation differ.

### DO PRE‐ AND POSTZYGOTIC ISOLATION EVOLVE AT THE SAME RATE?

C&O (1989) also compared the rates of evolution of premating and postzygotic isolation in order to “*know which type of isolation is most important in reducing gene flow between incipient species”* (page 363). Their analysis demonstrated that premating RI accumulates faster than postzygotic RI, especially in recently diverged species (Fig. 5 in C&O). Unlike the first prediction, that RI should increase with genetic divergence, few explicit tests have compared the rates of evolution of different types of barriers to gene flow. Besides C&O (1989) we found only eight studies (nine clades) that have compared the rate of evolution of different RI barriers (Table 1); premating RI evolved faster than postzygotic RI in five of the examined taxa (*Etheostoma* darters, *Drosophila*, *Gasterosteus* sticklebacks, food‐deceptive orchids, and sexually deceptive‐orchids; Table 1). The analysis of *Strepanthus* jewelflowers is notable because it is the first attempt to compare the rate of accumulation of ecological divergence (scored as climatic variables) with premating (phenology and floral distance), PMPZ (fruit set), and postzygotic traits (seed‐set success, seed mass, and F1 survival to flowering; Christie and Strauss 2018), demonstrating that niche differences tend to be stronger than other barriers in early stages of divergence. In *Cyrtodiopsis* stalk‐eyed flies, hybrid sterility evolves faster than premating and other types of RI (Charistianson et al. 2005), and in *Nolana* bellflowers postzygotic RI is stronger and evolves faster than prezygotic isolation (Jewell et al. 2012). In three more instances (*Silene* and *Glycine*, Moyle et al. 2004; and food‐deceptive orchids, Scopece et al. 2007, Scopece et al. 2008) prezygotic and postzygotic RI accumulate at similar rates. Thus, and although the plurality of studied taxa shows faster prezygotic than postzygotic RI, this pattern is not universal.

**Table 1:** Studies that have compared the rate of evolution of different RI barriers

**Taxon**	**Number of species pairs**	**Result**	**Phylogenetic correction**	**Reference**
Darters *Etheostoma*	13	Sexual isolation accumulates faster than postzygotic RI. Hybrid inviability does not increase over time.	Species pair selected to be strictly phylogenetically independent	(Mendelson 2003)
Stickleback *Gasterosteus*	5	Sexual and ecological isolation accumulate faster than other barriers	No	(Lackey and Boughman 2017)
*Streptanthus*	39	Climatic niche differences are substantial early in speciation and evolve faster than differences in phenology and floral morphology.	Node‐weighted average phylogenetic correction, (Fitzpatrick 2002)	(Christie and Strauss 2018)
*Nolana*	15‐22	Prezygotic pollen–pistile isolation accumulates slower than all postzygotic barriers.	Node‐weighted average phylogenetic correction, (Fitzpatrick 2002)	(Jewell et al. 2012)
*Drosophila*	72	Prezygotic and postmating‐prezygotic RI accumulate faster than postzygotic barriers.	Random sampling of strictly phylogenetically independent species pairs and bootstrapping	(Turissini et al. 2018)
*Glycine*	18‐55	Postmating‐prezygotic and postzygotic RI accumulate at similar rates.	Random sampling of strictly phylogenetically independent species pairs and bootstrapping	(Moyle et al. 2004)
*Silene*	19‐49	Postmating‐prezygotic and postzygotic RI accumulate at similar rates.	Random sampling of strictly phylogenetically independent species pairs and bootstrapping	(Moyle et al. 2004)
Food‐deceptive orchids	110, 125	Premating and postmating‐prezygotic RI show no clear increase over divergence. Postzygotic RI evolves in a clock‐like manner.	Node‐weighted average phylogenetic correction, (Fitzpatrick 2002) and identification of strictly phylogenetically independent species pairs	(Scopece et al. 2007)
Sexually deceptive orchids	36	Strong premating RI but weak postmating RI	Node‐weighted average phylogenetic correction, (Fitzpatrick 2002) and identification of strictly phylogenetically independent species pairs	(Scopece et al. 2007)
Stalk‐eyed flies	12	Hybrid male sterility accumulates faster than premating RI, hybrid inviability, and female hybrid sterility	No	(Charistianson et al. 2005)

Even though most studies have focused on comparing rates of evolution of prezygotic and postzygotic RI, other traits likely influence how species form and persist. Moyle et al. (2004) presented the first comparative study of RI in plants, and by doing so carried out the first analyses of PMPZ traits (post‐pollination prezygotic traits in the case of plants). For both *Glycene* and *Silene*, PMPZ and postzygotic traits evolve at similar rates (Moyle et al. 2004). Four studies have addressed the rate of accumulation of PMPZ barriers. The evolution of PMPZ and postzygotic RI in orchids varies across groups (Scopece et al. 2008). Postzygotic RI increases with divergence in food‐deceptive orchids, but not in sexually‐deceptive ones, but PMPZ does not increase with genetic distance for either type. In *Streptanthus* and *Nolana*, fruit set, a metric of whether pollen is able to germinate down the style and fertilize ovules, is not correlated with genetic distance (Jewell et al. 2012; Christie and Strauss 2018). Finally, in *Drosophila*, PMPZ RI evolves almost as fast as premating RI (Turissini et al. 2018). Differences among the rates of evolution of PMPZ RI in these few divergent taxa highlight the need for additional sampling to better understand the contribution of PMPZ barriers to species persistence as divergence increases.

More generally, premating barriers accumulate fast in multiple taxa. Yet, in other taxa, postzygotic RI accumulates as fast or faster than prezygotic RI. In the case of hybrid zones, some have argued that premating isolation is the most effective mechanism in keeping species apart (e.g., Kirkpatrick and Ravigné, Jiggins et al. 2001), while others argue that premating isolation alone is ineffective at maintaining species boundaries (Irwin 2020). In all likelihood, premating, PMPZ, and postzygotic RI act in conjunction to maintain species boundaries in nature (Servedio and Saetre 2003; Widmer et al. 2009; Schemske 2010), and the relative importance and order of appearance of different barriers will vary across taxa.

### DO HYBRID STERILITY AND INVIABILITY EVOLVE AT THE SAME RATE?

C&O (1989) next asked whether hybrid sterility and inviability evolve at different rates. This seems plausible given that hybrid sterility and inviability need not share genetic and/or developmental bases (Orr 1993, Sawamura 2000, Bundus et al. 2018; but see Barbash and Ashburner 2003, Sawamura et al. 2014), and that mechanisms of postzygotic RI may have a complex genetic basis (Matute et al. 2014; Phadnis et al. 2015; Barnard‐Kubow and Galloway 2017; Larson et al. 2018). Using a similar approach that they used to compare rates of evolution of prezygotic and postzygotic RI, C&O (1989) found that hybrid sterility and inviability evolve at similar rates, suggesting that these barriers are “*by‐products of similar genetic processes*” and equally likely to underlie the persistence of incipient *Drosophila* species. However, more recent work found that sterility accumulates faster than inviability (Wu 1992; Coyne and Orr 1997; Turissini et al. 2018). This discrepancy seems to result from how the data are analyzed, with average time of divergence between species pairs being an underpowered measure to assess differences in rates of evolution of different types of RI (Wu 1992; Coyne and Orr 1997; Coyne and Orr 2004, p. 75).

Five additional cases in non‐*Drosophila* taxa provide support for faster evolution of sterility than inviability. Using data collected on mammals (Gray 1972), Wu (1992) found 25 instances of Haldane's rule for sterility, but no good cases of Haldane's rule for inviability. Similarly, the mean age of Lepidopteran (Presgraves 2002), bird (Price and Bouvier 2002), and frog (Sasa et al. 1998) species showing hybrid sterility is lower than the mean age of species showing hybrid inviability. The rate of evolution of complete hybrid sterility is higher than the rate of accumulation of embryo mortality in food‐deceptive Mediterranean orchids (Scopece et al. 2008); and in *Cyrtodiopsis* stalk‐eyed flies, male hybrid sterility accumulates faster than hybrid inviability, hybrid female sterility, and premating RI (Charistianson et al. 2005). This rapid evolution of hybrid sterility in stalk‐eyed flies has been interpreted as evidence of pervasive genetic conflict (Charistianson et al. 2005). Introgression analyses in *Drosophila* have suggested that regions that cause hybrid sterility are much more abundant than regions that cause hybrid inviability (True et al. 1996; Masly and Presgraves 2007). Even though to date all studied taxa show evidence of faster evolution of hybrid sterility than inviability, given the small number of comparative studies addressing the relative rates of accumulation of different postzygotic barriers, the question of whether sterility evolves faster than inviability seems far from settled. Even if inviability evolves relatively slowly, it is worth noting that changes to the regulation of morphological development can evolve rapidly (Abzhanov et al. 2004; Shapiro et al. 2004; Mallarino and Abzhanov 2012), and hybrid inviability appears earlier in some taxa (e.g., mammals) than others (e.g., birds) (Prager and Wilson 1975; Fitzpatrick 2004), due in part to parent‐of‐origin‐dependent abnormal growth (Vrana et al. 2000; Ishikawa et al. 2011; Brekke and Good 2014; Rebernig et al. 2015; Oneal et al. 2016; Coughlan et al. 2020).

Both sterility and inviability are developmental defects that can manifest at different stages of development (Cutter and Bundus 2020). Analyses of gene expression across development suggest an “hourglass” model with increased divergence at intermediate developmental stages (Cruickshank and Wade 2008; Kalinka et al. 2010; Liu et al. 2019). To our knowledge, no analysis of gene expression across hybrid development exists, but the hourglass model predicts incompatibility to arise at early and late stages. Only two studies have dissected components of hybrid inviability across development. Closely related *Bufo* toad species produce hybrids that are more likely to reach later developmental stages than hybrids produced by more diverged pairs, which suggests that hybrid inviability at later stages of development evolves slower (Malone and Fontenot 2008). In *Drosophila*, embryonic inviability evolves before larval or pupal inviability (Turissini et al. 2018), and mapping of *X*‐linked incompatibilities reveals a higher number of embryonic and pupal incompatibilities than larval incompatibilities in hybrids between diverged species (Matute and Gavin‐Smyth 2014).

It is worth noting one important caveat about the comparison of the rate of accumulation of inviability and sterility (or between different developmental stages). Because sterility can only occur in viable hybrids, the range of genetic distances in which we can observe sterility is necessarily smaller than the range for inviability (Wu 1992; Coyne and Orr 2004 pp. 57–60). Similar issues also apply to the study of other traits; for example, in cases where behavioral isolation is complete, collecting information on the strength of postzygotic RI is unfeasible (but see Sánchez and Santamaria 1997). In summary, and in contrast to the original C&O (1989) result, current evidence suggests that hybrid sterility accumulates faster than inviability, but more work in additional taxa are needed to settle this question.

### HOW DOES POSTZYGOTIC ISOLATION INCREASE WITH TIME?

C&O (1989) built on their analysis of rates of hybrid sterility and inviability evolution by asking if these barriers increase with divergence time, and if so, whether they accumulate at different rates between the sexes. Analyses of postzygotic RI have generally found that in dioecious species, the sterile or inviable sex tends to be the heterogametic sex, a pattern known as “Haldane's rule” (Haldane 1922). Multiple genetic mechanisms for Haldane's rule have been proposed (reviewed in Wu et al. 1996; Laurie 1997; Orr 1997; Schilthuizen et al. 2011; Delph and Demuth 2016) and supported (Orr 1993; Masly and Presgraves 2007). There are relatively few exceptions, making Haldane's rule one of the only speciation “rules”. Indeed, of 223 cases of hybrid sterility in dioecious animals, 213 follow Haldane's rule. Of 452 cases of hybrid inviability, also in dioecious animals, 381 follow Haldane's rule (Table 2 in Schilthuizen et al. 2011). The rule applies to animal species with heteromorphic and homomorphic sex chromosomes (Presgraves and Orr 1998). C&O (1989) tested whether Haldane's rule appears early in the speciation process by evaluating genetic divergence between species that produce sterile or inviable hybrid males, sterile or inviable females, or sterility and inviability in both sexes. One would expect that if Haldane's rule is common it must necessarily precede the case where both sexes are sterile or inviable. Of the 21 pairs of recently diverged species, they evaluated after phylogenetic corrections, 19 produced hybrids whose sterility and inviability is limited to the heterogametic sex. The results suggest that male sterility and inviability evolve prior to hybrid female defects (Coyne 1989; Coyne and Orr 1997; but see Turissini et al. 2018).

A follow‐up approach in *Drosophila* found that in the *melanogaster* species complex, defects pertaining to males accumulate faster than those of females. Hybrid male inviability evolves faster than hybrid female inviability, and hybrid male sterility evolves faster than hybrid female sterility (Turissini et al. 2018). Contrary to the C&O (1989) findings, female sterility seems to evolve at lower genetic distances than male inviability (Turissini et al. 2018). Systematic introgressions between *Drosophila* species have revealed a higher number of hybrid male sterility alleles than of hybrid female sterility alleles (True et al. 1996; Sawamura et al. 2000; Masly and Presgraves 2007). Outside *Drosophila*, few studies have addressed which hybrid defects accumulate faster in the heterogametic sex. In birds, male F1 sterility appears earlier than female inviability. Cases of Haldane's rule for sterility are five times more common than for inviability (Price and Bouvier 2002; Arrieta et al. 2013). In Lepidopterans, hybrid female inviability often evolves prior to hybrid male sterility (Presgraves 2002). Additional analyses of when and how Haldane's rule appears during speciation are needed.

In species with chromosomal sex determination, comparative studies of the effects of sex chromosomes on interspecific hybrid fitness suggest that large sex chromosomes accumulate more hybrid incompatibilities than do smaller sex chromosomes. *X*‐linked incompatibilities often underlie intrinsic postzygotic isolation and Haldane's rule in *Drosophila* (Orr 1993; Good et al. 2008; Presgraves 2008, 2018; Meiklejohn and Tao 2010; Muirhead and Presgraves 2016), and *Drosophila* species pairs with relatively larger *X* chromosomes evolve Haldane's rule faster (Turelli and Begun 1997). Lepidopterans show a categorically different pattern. Despite having small sex chromosomes (comparable with *Drosophila* species with small *X*‐chromosomes; Traut et al. 2007; Kaiser and Bachtrog 2010), Haldane's rule for sterility appears relatively early in Lepidopterans (Presgraves 2002). No similar study has addressed whether heterosomes (i.e., sex chromosomes only present in the heterogametic sex) also correlate with RI. The collective observations of the accumulation of hybrid defects along divergence suggest that Haldane's rule is a common phase in the speciation process.

More generally, the presence of sex chromosomes has been hypothesized to contribute to faster evolution of postzygotic RI (Johnson 2010; Johnson and Lachance 2012; Phillips and Edmans 2012). The evidence for this hypothesis is limited and comes from three sources. First, haplodiploid species, which do not have differentiated sex chromosomes but show Haldane's rule, seem to evolve hybrid incompatibility more slowly than other insects (Koevoets and Beukeboom 2009). Second, using diversification rates and species richness from Eo and DeWoody (2010), Phillips and Edmands (2012) concluded that lizards and snakes (squamates) which have differentiated sex chromosomes have speciated more quickly than turtles and crocodilians, two clades in which differentiated sex chromosomes are rare and absent, respectively. This pattern is not followed by birds that universally possess ZW sex. Finally, a meta‐analysis of 26 species pairs suggests that taxa without sex chromosomes show the slowest rates of evolution of postzygotic RI, but the effect of the presence of sex chromosomes is small (Lima 2014). Sex chromosome turnover is also associated with the evolution of intrinsic postzygotic RI in stickleback fish (Kitano et al. 2009; Kitano and Peichel 2012) and bark beetles (Bracewell et al. 2017). Whether sex chromosomes (including the evolution of neo‐sex chromosomes) and dioecy accelerate the accumulation of RI and speciation rates remains unknown.

### IS PREZYGOTIC RI ENHANCED BY SELECTION WHEN POPULATIONS BECOME SYMPATRIC?

Finally, C&O (1989) examined if RI accumulates faster in sympatry versus allopatry. Under this hypothesis known as reinforcement, natural selection may accelerate the speciation process by penalizing maladaptive hybridization, favoring stronger prezygotic RI to avoid maladaptive hybridization in species with overlapping geographical ranges (Liou and Price 1994; Servedio and Noor 2003; Schlichting and Mousseau 2009; Hopkins 2013). Potential costs of hybridization include hybrid defects and costs to females after heterospecific matings (Servedio 2001, 2011; Lorch and Servedio 2005). Importantly, for *Drosophila* and other species with internal fertilization and external development, only prezygotic RI should be reinforced because selection does not act to increase postzygotic RI in sympatry (Wallace 1889, Ch. 7; Coyne 1974). Multiple cases reported the possibility of reinforcement before C&O (1989) (e.g., Littlejohn 1965; Fouquette Jr 1975; Loftus‐Hills 1975; Markow 1981; Levin 1985). For example, Ehrman (Ehrman 1965) found the first strong support for reinforcement, demonstrating greater premating isolation for most comparisons of sympatric populations of “semispecies” in the *D. paulistorum* clade (Dobzhansky and Spassky 1959). However, the first large‐scale comparative study to lend evidence to the possibility of pervasive reinforcement across a large taxonomic group was C&O (1989).

To test for reinforcement, C&O (1989) regressed the magnitude of RI on the genetic distance between sympatric and allopatric *Drosophila* species pairs, independently. In the case of both prezygotic and postzygotic RI, the intercept should be similar as recently diverged species have low costs to hybridization. In contrast, if reinforcement has taken place, RI should accumulate more quickly for sympatric than for allopatric pairs, generating differences in the regression slopes. This is precisely what C&O (1989) found—the mean degree of premating RI, but not postzygotic RI, is twice as large for sympatric species than for allopatric species. This was the first evidence widely supporting reinforcement over competing hypotheses (e.g., differential fusion and extinction; Templeton 1981; Butlin 1987), and served to fuel a body of research on the role of reinforcement in speciation in other systems (Noor 1995; Niet et al. 2006; Hopkins and Rausher 2012; Castillo and Moyle 2019).

In the last 30 years, similar tests have assessed whether reinforcement is pervasive in other clades, and the evidence is mixed. In fungi, homobasidiomycota show a faster accumulation of total RI in sympatric species pairs than in allopatric pairs (Giraud and Gourbière 2012). Evidence for reinforcement also exists in plants (Niet et al. 2006; Grossenbacher and Whittall 2011), although as noted by Hopkins (2013) uncertainty about the role of reinforcement in plant speciation remains due to both approximate and incomplete measures of RI. Comparing RI in sympatric and allopatric species has shown no evidence of reinforcement in *Glycine* and *Silene* plants (in either postmating prezygotic or postzygotic, Moyle et al. 2004), ascomycetous fungi (in total isolation, Giraud and Gourbière 2012), or doves (Lijtmaer et al. 2003). Clearly, even if reinforcement is a common step in the completion of speciation, it does not leave a universal signature in sympatric species.

The use of the comparative approach also propelled new tests, all of which are framed within the use of phylogenetically corrected RI datasets. A second test involves identifying triads of species for which the magnitude of RI is known. If one of the species pairs is sympatric and the other allopatric, and reinforcement has acted on the sympatric pair, then the magnitude of RI should be stronger in the sympatric pair (Noor 1997). In spite of the power of this approach triads of species have only been used to infer reinforcement of behavioral isolation in *Drosophila* (Turissini et al. 2015), bird plumage (Martin et al. 2015), bird body size (Bothwell et al. 2015), and bird chromosomal rearrangements (Hooper and Price 2017). This latter trait might be associated with the likelihood that species in sympatry persist when they have the chance to hybridize, and not associated with reinforcement itself (e.g., Hooper et al. 2019).

A third test using comparative data to infer reinforcement involves identifying species pairs with asymmetric levels of premating and postzygotic RI (Yukilevich 2012). In cases with strong postzygotic RI, the influence of reinforcing selection should be strong due to a high risk of maladaptive hybridization. Throughout nature, the fitness of hybrids produced by reciprocal crosses often differs (Darwin 1859, Ch. 8), generating a pattern of asymmetrical postzygotic RI known as “Darwin's corollary to Haldane's rule” (Turelli and Moyle 2007). In these cases, reinforcing selection should generate elevated prezygotic RI for the side of the cross with stronger postzygotic RI. Yukilevich (Yukilevich 2012) tested this hypothesis and found a pattern of concordant asymmetries is more common in sympatry than in allopatry. This pattern could be caused by reinforcement, or by other processes that favor the co‐occurrence of premating and postzygotic RI simultaneously. For example, asymmetries in gene flow could encourage the asymmetric accumulation of intrinsic postzygotic RI (Turelli et al. 2014). An extension of this approach uses the proportion of overlapping geographic range as a proxy for the risk of hybridization (Nosil 2013). Species pairs that share a larger proportion of their geographic range are more likely to hybridize and thus the proportion of geographic range that two hybridizing species share should be proportional to their RI. Indeed, there is a negative correlation between the strength of premating isolation and geographic overlap which has been interpreted as additional evidence for the role of reinforcement on speciation.

The relative frequency of reinforcement remains a contentious question in speciation (Hudson and Price 2014; Turelli et al. 2014). Reinforcement debates prior to C&O (1989) were mostly centered around whether reinforcing selection is plausible (Paterson 1978; Spencer et al. 1986). Since C&O (1989), the question has been repeatedly re‐evaluated to assess the relative importance of this process in nature. Some have argued that reinforcement is pervasive (Hudson and Price 2014); and given the ubiquity of sympatry in *Drosophila* (Nosil 2013; Turelli et al. 2014), and the possibility of recapitulating reinforced RI in the laboratory with experimental evolution (Koopman 1950; Rice and Hostert 1993; Etges 1998; Higgie et al. 2000; Matute 2010a,b), Turelli et al. (2014) concluded that reinforcement must be common, at least in *Drosophila*. While we have no definitive answers yet on how frequently reinforcement acts in speciation, C&O (1989) opened the door to study it in a systematic and comparative manner.

## Future Questions

By definition, speciation is at the interface of microevolutionary and macroevolutionary processes. C&O (1989) posed and addressed five questions using the comparative approach. These research avenues are still priorities and represent a large portion of the current speciation research. Still, the field has changed considerably since 1989, and two specific developments seem worth mentioning. First, the infusion of genomic data has revitalized speciation research, giving rise to new hypotheses and methods to test them (Butlin 2010; Rice et al. 2011; Seehausen et al. 2014; Campbell et al. 2018). Second, the field has incorporated robust population genetics and phylogenetic methods to understand diversification over time (reviewed in O'Meara 2012; Pennell and Harmon 2013). The combination of these data and methodological advancements has generated new questions and challenges that can be addressed with variations of the comparative approach. Here, we list five directions that the field is poised to address using a combination of comparative phylogenetic methods, genomics, and natural history.

### THE AMOUNT OF INTROGRESSION AS DIVERGENCE PROCEEDS

The converse aspect of the scaling of RI with divergence is that the amount of gene exchange between sympatric taxa should decrease as genetic divergence accrues for two reasons. First, hybrid production decreases as divergence accrues, which reduces the possibility for hybrids to serve as a bridge for gene exchange between parental species. This is supported by the reduction in the number of naturally occurring hybrids as divergence increases (e.g., Mallet 2007; Pereira et al. 2011; Sánchez‐Guillén et al. 2014). Second, hybrids produced by more divergent parents will have more hybrid incompatibilities, thus purging introgression even if hybridization occurs (Orr 1995; Matute et al. 2010; Moyle and Nakazato 2010; Wang et al. 2015). Notably, the decrease in gene exchange might be precipitous across the ‘gray zone of speciation’—the level of differentiation in which species definition is controversial (Roux et al. 2016). Few tests for a relationship between amounts of introgression and the divergence between species pairs exist, but the magnitude of segregating introgression in *Phletodon* salamander (Wiens et al. 2006)*, Heliconius* butterfly (Kronforst et al. 2013), and *Solanum* tomato (Hamlin et al. 2020) species pairs is inversely correlated with the age of divergence of the hybridizing species. Whether this is a pattern that applies to other taxa remains unknown.

### THE EFFECT OF GENETIC DISTANCE ON HYBRID SPECIATION

Hybrid speciation remains a relatively rare and highly controversial form of speciation (Schumer et al. 2014; Schumer et al. 2018; Nieto Feliner et al. 2017). In this process, hybrid populations become reproductively isolated from the parental forms as a result of admixture (Gross and Rieseberg 2005; Mallet 2007). Since the possibility of hybrid speciation is contingent on the fitness of hybrids from early generations, genetic distance is a factor that might determine the likelihood of the process. Hybridization could fuel adaptive radiations, but how the genetic distance between hybridizing species influences the likelihood of hybrid speciation remains largely understudied (Seehausen 2004; Meier et al. 2017; Marques et al. 2019). Multiple theoretical models have proposed mechanisms on how hybrid speciation might proceed (Buerkle et al. 2000; Schumer et al. 2015; Blanckaert and Bank 2018; Comeault 2018; Yamaguchi and Otto 2020), but few empirical studies exist. In plants, the likelihood of allopolyploidy depends on the magnitude of genetic divergence (Chapman and Burke 2007; Paun et al. 2009); and in experimentally produced, admixed populations of *Drosophila* that differed in their degree of genetic relatedness (Comeault and Matute 2018), hybrid swarms from parents with intermediate levels of divergence were most likely to become reproductively isolated from the parental species. These two results suggest that intermediate levels of divergence might be more permissive for speciation by hybridization and that, as proposed by the theoretical models, there might be a level of genome divergence that facilitates hybrid speciation. As is the case with the relative prevalence of hybrid speciation in evolution, the importance of genomic traits such as recombination rates, presence of sex chromosomes, and chromosomal rearrangements for the formation of hybrids species remains largely unknown.

### COMPARATIVE RATES OF EVOLUTION ACROSS DIFFERENT TAXA

RI could limit rates of diversification across taxa (Mayr 1963). If so, taxa should vary in their rate of RI acquisition, and groups with a greater propensity to evolve RI should become more speciose. This question precedes C&O (1989) and remains unsolved today. Seminal work revealed that hybrid inviability evolves faster in mammals than in birds and frogs, which was attributed to rapid regulatory evolution in mammals (Wilson et al. 1974; Prager and Wilson 1975; Fitzpatrick 2004). More recent taxon‐specific comparisons have demonstrated that postzygotic RI evolves faster in *Drosophila* and in Lepidoptera than in anurans (Russell 2003; Mendelson et al. 2004). Few studies have formally tested whether taxa displaying faster RI evolution also harbor more species. Meta‐analyses have failed to find a relationship between the strength of assortative mating and species richness (Janicke et al. 2019). New comparative phylogenetic methods have revitalized this question. Nodal‐network models suggest that RI from pairwise crosses can be used to estimate the strength of RI as an individual species trait (i.e., whether a single species is more prone to being isolated from all other species). *Drosophila* and bird species differ significantly in their RI as a species trait—some species evolve RI more quickly than others (Rabosky and Matute 2013). Similar results, in terms of the per branch speciation rate, have been reported in lizards (Singhal et al. 2018). Notably, there is no correlation between the rates of RI acquisition and diversification rates in *Drosophila* or birds. Together, these results suggest that the rate of RI evolution might not be the factor limiting the evolution of new species. In turn, the persistence of species when they have the chance to hybridize (and go extinct) might play an important role in determining species richness (Rosenblum et al. 2012; Harvey et al. 2019). This direction will benefit from a systematic study of RI across taxa, the generation of robust time‐calibrated phylogenies, and the collection of generation times, which will jointly enable modeling RI under various models of trait evolution (Moyle and Payseur 2009; Huey et al. 2019).

### MACROEVOLUTIONARY METRICS OF DIVERSIFICATION

RI might also be associated with global patterns of biodiversity (Mayr 1963; Rabosky 2016). There are more species in the tropics than in temperate areas, which generates questions about the relative contributions of speciation and extinction rates to this pattern, including how they vary spatially (reviewed in Willig et al. 2003; Schemske et al. 2009; Jablonski et al. 2017). The rates of evolution of traits that differentiate species vary clinally with latitude. In birds, climatic‐niche (Lawson and Weir 2014) and bird song traits (Weir and Wheatcroft 2011) evolve faster at higher latitudes, yet studies comparing rates of RI acquisition in tropical and temperate species are rare. Only one study has tested whether RI evolves faster between tropical than between temperate species. Yukilevich (Yukilevich 2013) found that, after controlling for genetic distance, species pairs in the tropics display stronger hybrid sterility than species in temperate areas. (This pattern does not exist for premating RI.) The connection between metrics of diversification and RI remains in its infancy, but it is one of the most urgent questions in speciation biology. Similar to the need for robust time‐calibrated phylogenies to understand whether clades differ in how RI accumulates, only a systematic study of the accumulation of RI across taxa and of species richness across time will reveal whether the acquisition of RI influences the rate of speciation at macroevolutionary scales.

### DARWIN'S COROLLARY TO HALDANE'S RULE

Reciprocal crosses often differ in the magnitude of RI as described above (Tiffin et al. 2001; Turelli and Moyle 2007; Lowry et al. 2008). While C&O (1989) did not address this pattern, theoretical models (Turelli and Moyle 2007) and a handful of empirical examples (Sawamura and Yamamoto 1993; Ferree and Barbash 2009; Sawamura et al. 1993a; Sawamura et al. 1993b) suggest that uniparentally inherited genetic factors play an important role in explaining differences in postzygotic RI between reciprocal crosses. These can include effects of maternally transmitted symbionts like *Wolbachia* bacteria on RI (Williamson and Ehrman 1967; Miller et al. 2010), although this is likely rare (Cooper et al. 2017). Parent‐of‐origin effects are common in taxa with placentas. Thus, the relative frequency of asymmetric RI might differ between groups that have a placenta and imprinting (e.g. mammals, angiosperms) and groups that do not (e.g. birds). Asymmetrical RI could have important consequences for how speciation is completed. Asymmetric gene exchange might reduce the likelihood of reinforcement and the likelihood of introgression (Servedio and Kirkpatrick 1997). Studies on the relationship between genetic distance and RI asymmetry are rare. As divergence accrues, asymmetry in RI increases in centrarchid fishes (Bolnick and Near 2005). There is no relationship in the asymmetry in ability to find food in *Drosophila* hybrids (Turissini et al. 2017) or in the asymmetry of isolation in *Jaltomata* nightshades (Kostyun and Moyle 2017). In general, even though asymmetries in RI are common and genetic models have been extensively researched, the consequences of Darwin's Corollary in speciation remain largely unknown.

## CONCLUSIONS

The seminal work of C&O (1989) demonstrated the power and utility of the comparative approach for understanding general patterns of RI, but there are limitations, including the inability of these analyses to reveal which barriers contribute most to speciation. The spirit of several analyses in C&O (1989) was to determine “*which type of isolation is most important in reducing gene flow between incipient species*.” Decades of cataloguing species differences and identifying traits that reduce gene flow strongly suggest that different traits function jointly to maintain species boundaries. These traits might evolve simultaneously and affect the rates of evolution of other traits (e.g., Langerhans et al. 2007). The existence of reinforcement itself confirms the complex interaction between different traits, as prezygotic RI may evolve as a byproduct of selection against maladaptive hybridization.

Since C&O (1989), hundreds of studies have described the increase of RI along divergence. Still today, there is not a unified systematic understanding of how RI accumulates in phylogenetic trees, and the infusion of a fully quantitative phylogenetic background to the study of RI remains in its infancy. Comparative approaches to study speciation remain relevant and will continue to inform how species form and persist in nature. We are optimistic about the future of speciation research, including the continued influence of C&O (1989) on the field.

## AUTHOR CONTRIBUTIONS

D.R. Matute and B.S. Cooper wrote the article.

Associate Editor: T. Chapman

Handling Editor: T. Chapman
